# Case report: Generalized lymphatic anomaly of multiple abdominal organs in a young dog

**DOI:** 10.3389/fvets.2023.1154210

**Published:** 2023-05-05

**Authors:** So-Hyeon Park, Jung-Ha Lee, Elisa N. Salas, Myeongsu Kim, Jae-ik Han, Kichang Lee, Hakyoung Yoon

**Affiliations:** ^1^Department of Veterinary Medical Imaging, College of Veterinary Medicine, Jeonbuk National University, Iksan-si, Republic of Korea; ^2^V Animal Medical Center, Jeju-si, Jeju-do, Republic of Korea; ^3^IDEXX Laboratories, Inc., Westbrook, ME, United States; ^4^Laboratory of Wildlife Medicine, College of Veterinary Medicine, Jeonbuk National University, Iksan-si, Republic of Korea

**Keywords:** lymphangiomatosis, lymphangioma, lymphatic malformation, multi-organs, canine, cystic lesions

## Abstract

A 9-month-old, female Pomeranian dog presented with vomiting and lethargy. Ultrasonography revealed multilobulated anechoic round shape structures at the ovarian and uterine locations. Through computed tomography scan, an extensive non-contrast multilobulated fluid-filled mass suspected of originating from the walls of the ovary, uterus, urinary bladder and rectum was observed. Ovariohysterectomy and urinary bladder biopsy were performed. Histopathological examination revealed numerous cystic lesions lined by plump cuboidal cells believed to be of epithelial origin. Immunohistochemical staining showed that the cyst-like lesions lining cells were strongly positive for lymphatic vessel endothelial hyaluronan receptor 1. Based on these results, lesions were identified as generalized lymphatic anomaly (GLA), in which lymphangiomas develop in multiple organs. After 6 months follow-up, the size of the cysts remaining in the region of the bladder did not undergo much change. GLA should be included in the differential diagnosis when multiple cystic lesions are interspersed in multiple organs.

## 1. Introduction

Lymphatic anomaly (lymphangioma, lymphatic malformation, lymphangiomatosis), a rare congenital disorder of the lymphatic system, is considered to arise from a failure of primitive lymphatic systems to adequately separate from or communicate with the venous system (1). These malformations cause dilation and proliferation of lymphatic vessels in animal & human.

Canine lymphatic anomalies have most commonly been reported in the skin, soft tissue, and retroperitoneum (2–7). It is seldom observed in parenchymal organs, and only two cases have been reported in the liver and spleen (8, 9).

In human, according to the International Society for the Study of Vascular Anomalies (ISSVA), Lymphatic anomlies can be divided into cystic lymphatic anomaly and complex lymphatic anomaly, depending on whether they occur in solitary lesions or multiple organs. Cystic lymphatic anomaly mainly occurs in the head, neck and extermities, and is divided into macro and micro depending on the size. Complex anomalies have been reported in <1% of humans and have not yet been reported in animals. It occurs in various organs such as various bones, liver, and spleen. There are three types of complex anomaly, GLA, Gorham-Stout disease (GSD), and Channel type lymphatic anomaly (CCLA), and there is a subtype of GLA, Kaposiform lymphangiomatosis (KLA). KLA is characterized by being surrounded by Kaposi-form cells, and mainly causes diseases in the chest. GSD is a disease that causes bone destruction and CCLA is a disease known as lymphangiectasia, which occurs when lymphatic vessels are damaged by obstruction of lymphatic vessels. GLA can also occur in bone, but it does not cause destruction (10, 11).

The clinical symptoms and prognosis of lymphatic anomaly varies depending on the locations of the affected organ and sizes of cystic lesions. Leakage of lymph or chyle may be intraperitoneal or intrathoracic, and hypoalbuminemia, hypoproteinemia, and lymphocytopenia may occur due to lymphangiectasia (11).

Lymphatic malformation is characterized by cystic lesions on Ultrasonography (US), Computed tomography (CT), and Magnetic resonance imaging (MRI). The existence and location of the cystic structures can be determined using US, and information about the range of lesions and adhesion of the surrounding tissues can be obtained through CT. In human, when GLA is suspected, MRI is used to identify abdominal and thoracic lesions and to identify mesenteric and retroperitoneal cyst-like lesions. MRI can evaluate soft tissues with good resolution and without radiation exposure. Also, MRI is known to be good for detecting minimal lymphatic lesions, and it is known to show hyper signal intensity in fat-saturated T2-weighted MRI and intense enhancement in gadolinium contrast agent (12–14).

However, immunostaining such as lymphatic vessel endothelial hyaluronan receptor 1 (LYVE-1), podoplanin, and Vascular endothelial growth factor receptor 3 (VEGFR-3) specific to lymphatic vessels is required for diagnosis. In humans, studies on genes related to lymphatic anomaly have been conducted, but no studies have been conducted in dogs yet (15, 16).

This is the first case of a dog with lymphatic anomaly in several parenchymal organs including ovary, uterus, intestine and bladder. Imaging diagnosis and immunohistochemistry was performed, and follow-up was performed 6 months later.

## 2. Case description

A 9-month-old Pomeranian dog presenting with vomiting and lethargy, was admitted to a local animal hospital. No abnormal findings were observed in body temperature, blood pressure, respiration rate, or pulse. Also, No remarkable abnormal findings were observed in the serum biochemistry panel and complete blood count.

**Figure 1:**
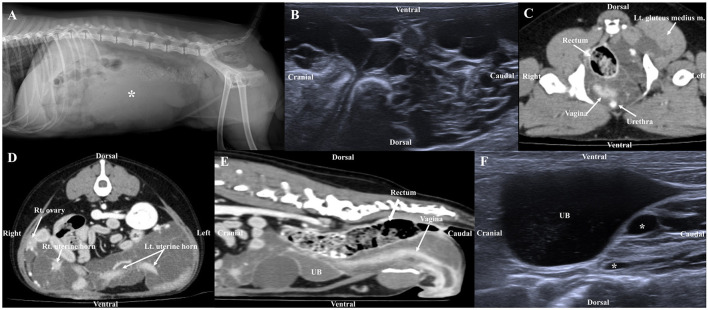
Radiographic image **(A)**, ultrasonography **(B, F)**, post-contrast CT **(C, D, E)** in a dog with lymphatic anomaly. **(A)** The tubular structure of soft tissue opacity (asterisk) in the caudoventral abdomen, **(B)** Ultrasonographic image shows multiple anechoic structures in the ovary and uterus, **(C)** Multilobulated cystic lesions originating from the left sublumbar lymph node expand through the pelvic canal to the left gluteus medius muscle, **(D, E)** Multiple cystic lesions were observed extensively in contact with the urinary bladder (UB), ovary, uterine, rectum, making it difficult to determine the origin and **(F)** Ultrasound image 6 months post-surgery, the size of cystic lesions remaining around the bladder was similar to that of 6 months prior.

Abdominal radiography (1417WGC, Rayence Co., Ltd., Hwaseong-si-, South Korea) was performed. In the lateral view, a tubular structure of soft tissue opacity with a size of 11.17 × 5.92 mm was observed in the ventral aspect of the descending colon in the caudoventral aspects of abdominal cavity at L1 to pelvis level. Therefore, the small intestine was cranially displaced (Figure 1A). In the chest radiography, increased opacity of the cranial lung lobe was observed in the lateral view, but this was considered to be due to summation of the muscles of the forelimb. In the VD view, the opacity of the left cranial lung lobe was increased, but the left deviation of the heart was observed, so atelectasis was considered first.

Abdominal US (ACUSON juniper, Siemens Healthineers, Erlangen, Germany) using 3.6–12.9 MHz linear transducers identified multiloculated anechoic structures with acoustic enhancement at the ovarian and uterine locations (Figure 1B), which was similarly observed near the urinary bladder (UB). Due to the size and range of the cystic lesion, the exact location could not be determined by US. The walls of the cysts were thin (<1 mm) and of various sizes and shapes with the largest being 2.37 × 2.27 cm. The boundary between the organs and cystic lesions was unclear due to displacement and obscuring by the structures.

CT was performed using a multislice CT scanner (TSX-031A; Toshiba Medical Systems Co., Ltd., Tochigi, Japan). The imaging parameters were as follows: 120 kVp, 200 mAs; matrix size, 512 × 512; rotation time, 0.75 s; and, slice thickness, 1 mm. For CT scans, the dog was induced with propofol (6 mg/kg, IV), and anesthesia was maintained by isoflurane. After obtaining the pre-contrast images, iohexol (750 mg iodine/kg) was manually injected into the cephalic vein. Postcontrast images were obtained after 120 s. Multilobulated structures were observed, starting from the vagina and extending to the uterine cervix and horns. The septa of the cystic structures were connected to the uterine wall, and the margin of the uterus was spiculated (Figures 1D, E). Therefore, the lesions were considered to originate from the uterine wall. In addition, they were observed to be in contact with the ventral aspect of the rectum, caudolateral aspect of the UB, both ovaries, part of the small intestine, and both sides of the anal sac, so the possibility of adhesion as the origin of the cystic structures could not be excluded. The lumens of multiple cystic lesions (up to 3.83 × 2.93 × 5.58 cm [L × H × W]) were observed with fluid attenuation (HU:13), and no significant enhancement was observed, but the walls showed significant enhancement. A multilobulated cystic structure similar to that observed in the uterus was also observed at the location presumed to be the left sublumbar lymph node, which expanded to the left gluteus medius muscle through the pelvic canal (Figure 1C). No bone lesion or ascites was observed around the mass, and no significant enlargement of the surrounding lymph nodes was observed. This mass was supplied with blood by the bilateral ovarian and vaginal arteries. No obvious abnormal findings were observed in the lung parenchyma and bronchus, and neither pleural effusion nor pneumothorax was observed.

Ovariohysterectomy and biopsy of the UB were performed to remove and diagnose the lesions. The dog was induced with propofol (6 mg/kg, IV), and anesthesia was maintained using isoflurane. Multiple cystic lesions originating from the walls of the uterine, ovary, urinary bladder, and intestine were widely observed, and cyst-like lesions and adjacent organs adhered to each other. Therefore, it was difficult to clearly demarcate and remove the lesions. After ventral median celiotomy, the ovarian pedicles and uterine body were clamped and ligated with polydioxanone. As complete resection of the cysts was impossible; debulking was performed. Tissues of the ovaries, uterine horns, and UB were fixed in 10% neutral-buffered formalin and sent for histological evaluation in a laboratory (IDEXX Laboratories Inc., Seoul, South Korea).

**Figure 2:**
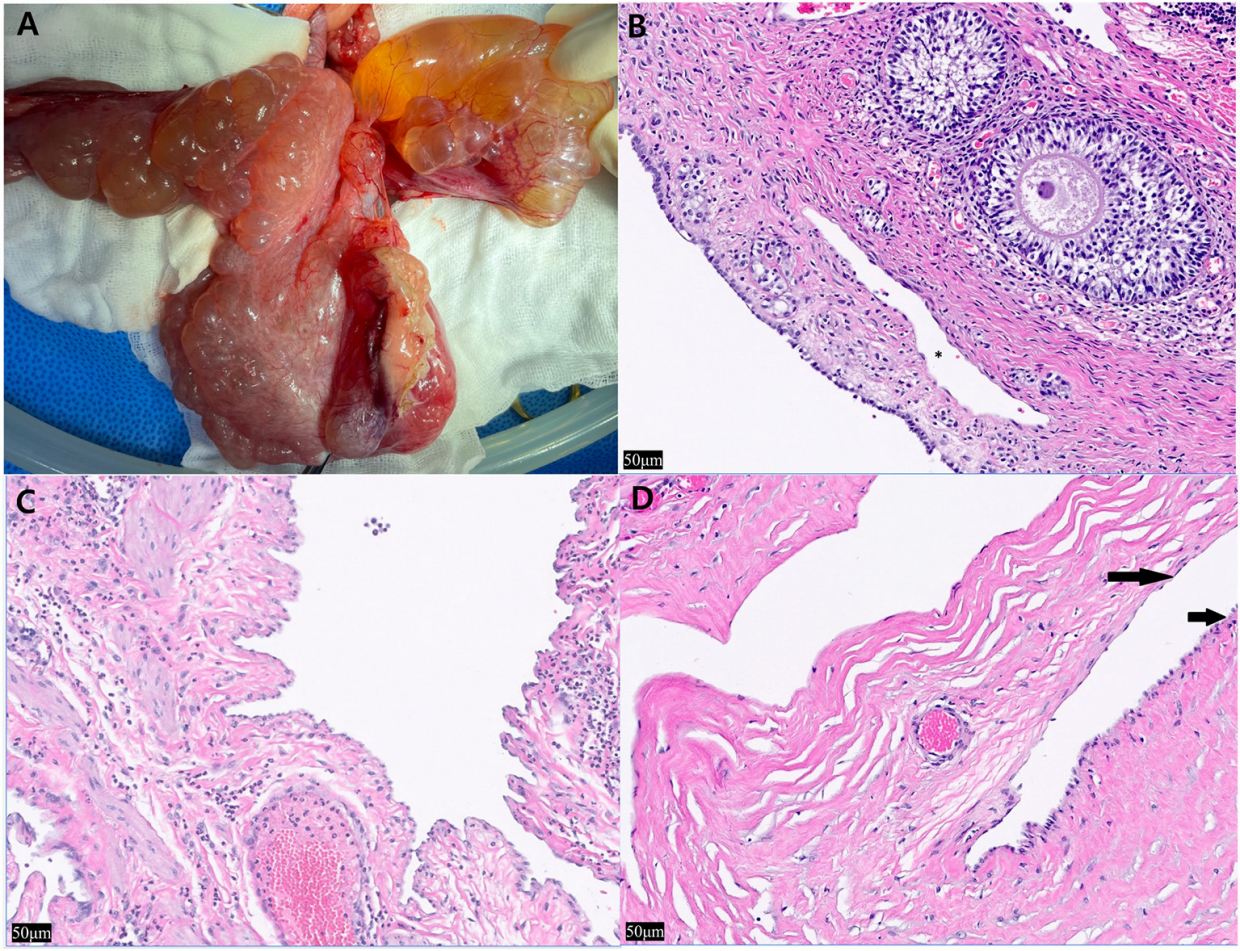
Intra-operative photographs of the ovary and uterus and histopathological findings after hematoxylin and eosin staining of the ovary, uterine horn, and urinary bladder. **(A)** The multiple thin-walled cystic structures expand the uterine smooth muscle, ovary and are see on the serosa. Cyst-like lesions and adjacent organs adhered to each other, so it was difficult to clearly demarcate and remove the lesions, **(B)** left ovary, approximately five cyst-like structures are seen peripheral to the ovum (*) (×20), **(C)** left uterine horn, the structures multifocally expand the myometrium. Most were empty and contained intraluminal pale eosinophilic proteinaceous material. Low to moderate numbers of neutrophils are noted bordering the vasculature with no associated necrosis, inflammation, or reactive vessels (×20). **(D)** Within the smooth muscle wall of the urinary bladder, the structures are lined by spindle cells (long arrow) and hypertrophied cuboidal cells (short arrow) (×20).

**Figure 3:**
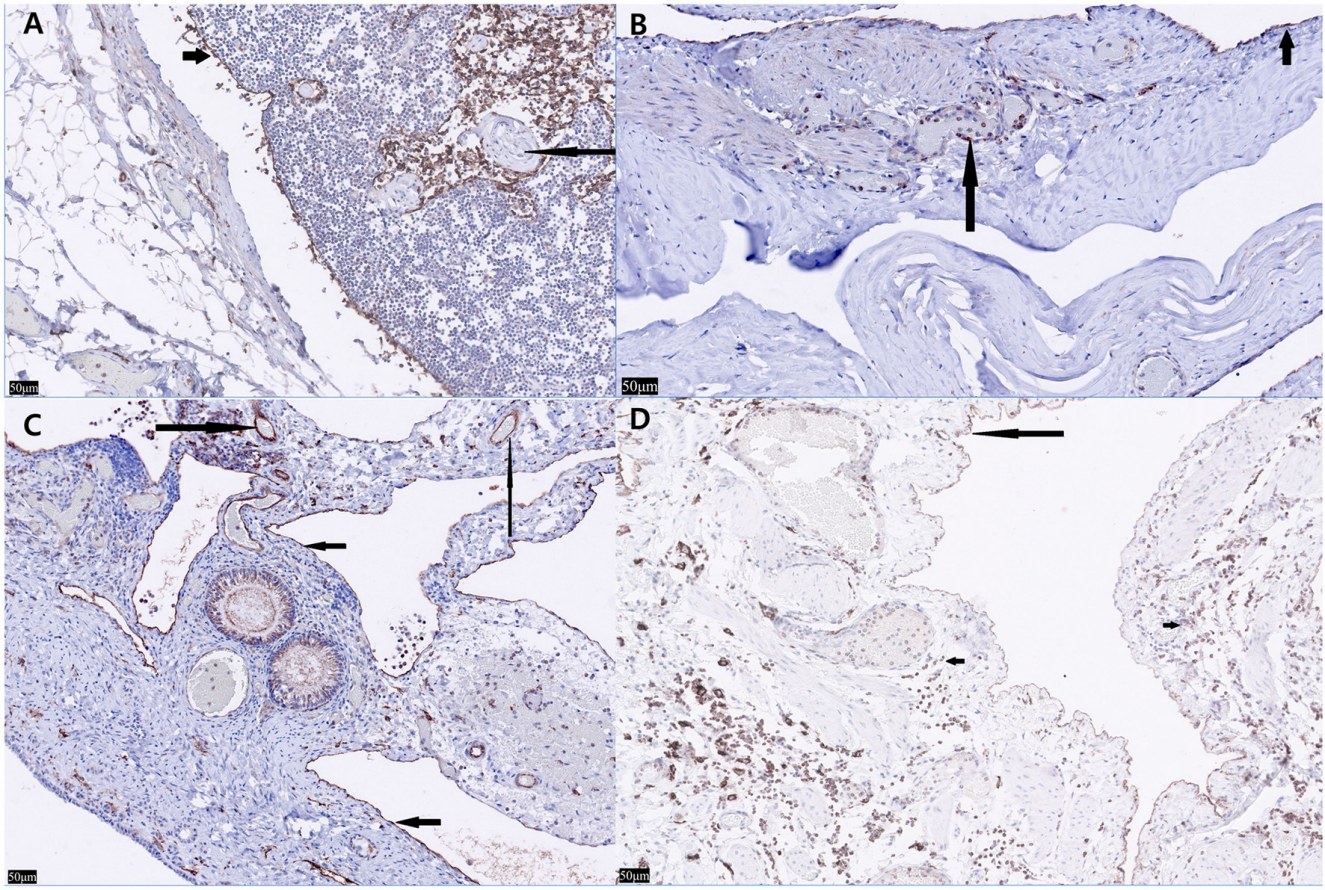
Lyve-1 immunostaining of the ovary, uterine horn, urinary bladder, and normal canine lymph node. **(A)** In the normal canine lymph node (control tissue), strong immunoreactivity is seen within lymphatics with nonspecific staining of leukocytes within the lymphatics (short arrow). Neither the arterioles nor interstitial leukocytes show immunoreactivity (long arrow) (×20). **(B)** In the urinary bladder, Lyve-1 reveals strong cytoplasmic immunoreactivity within the spindle cells both lining the cystic spaces (short arrow) and within the endothelial cells lining blood-filled vascular spaces (long arrow) (×20). **(C)** In the left ovary, Lyve-1 reveals strong cytoplasmic immunoreactivity lining the cystic spaces (short arrows). Intermediate immunoreactivity is also noted within endothelial cells lining blood-filled vascular spaces (Long arrows) (×20). **(D)** In nonspecific staining of leukocytes is seen (short arrows) in the left uterine horn, cells lining the cystic spaces show intermediate to strong immunoreactivity to Lyve-1 (long arrow) (×20).

Grossly, multiple pale yellow, clear-to-serous cystic structures were observed in the ovary and uterus (Figure 2A). Histological examination of the three organs confirmed numerous cystic structures lined by spindle to cuboidal cells. The ovary contained cyst-like lesions within the mesovarium and the ovarian parenchyma (Figure 2B). The uterus exhibited cyst-like leisons penetrating the myometrium (Figure 2C). The UB contained cystic structures within its smooth muscle wall (Figure 2D). Immunostaining using LYVE-1 in the ovary, uterus, and bladder tissue was performed to confirm whether it was derived from the lymphatic system. The cystic structures were Pancytokeratin AE1/AE3 negative throughout all the tissue, ruling them out as classically defined cysts: expansile spaces lined by epithelium. Rather, they exhibit intermediate to strong cytoplasmic immunoreactivity for LYVE-1, supporting endothelium lined spaces supportive of GLA (Figures 3A–C). The control tissue showed strong immunoreactivity in the lymphatics, but the arterioles showed no immunoreactivity (Figure 3A). However, intermediate immunoreactivity is also noted within endothelial cells lining blood-filled vascular spaces (Figures 3B, C).

DNA was extracted from the formalin-fixed and paraffin-embedded (FFPE) samples through the MagMAX™ FFPE DNA/RNA Ultra Kit (Thermo Fisher Scientific, Waltham, MA, USA) and QIAamp DNA FFPE Tissue Kit (Qiagen, Hilden, Germany) for Neuroblastoma RAS viral oncogene homolog (NRAS) gene sequencing. The NRAS coding region was amplified twice using forward (TACAAACTG GTGGTGGTTGG) and reverse (TCCAACAGACAGGTTTCACC) primers by HotStarTaq master mix kit (Qiagen, Hilden, Germany).

As a result, the peak size of the our patient was observed to be 44 base pairs both the times, instead of 150 base pairs with NRAS gene in a normal dog.

At the 6-months follow-up, the vomiting had disappeared and no significant change in the size of the cystic lesions remaining in the region of the bladder was seen (Figure 1F).

## 3. Discussion

In human medicine, lymphangiomatosis refers to lymphatic anomaly occuring in multiple organs and is known as CLA. GLA, one of the CLAs, is distinguished from other types like GSD, CCLA in that there are no bone destruction and no leakage of lymph (10, 11). Our patient was considered GLA because lymphatic anomaly occurred in multiple organs and there were no ascites or bone lesions. Since the previously reported canine lymphangiomatosis was a case of multiple cysts in one organ rather than multiple organs (3, 9, 17, 18), the use of the term lymphangiomatosis may cause confusion as to whether it is a single organ or multiple organs. So the authors referred to our case as GLA.

Lymphatic anomalies in humans, most commonly occurs in the neck (75%) and axillary sites (20%) and have rarely been reported in the mediastinum, omentum, mesentery, retroperitoneum, colon, pelvis, and bone. Less than 1% of cases are reported in internal organs, and involvement of three or more intra-abdominal organs is extremely rare in humans (19). Canine lymphatic anomalies have most commonly been reported in the skin, soft tissue, and retroperitoneum (2–6). It is seldom observed in parenchymal organs, and only two cases have been reported in the liver and spleen (8, 9). There have been no reports of urinary bladder, ovary, uterus, or rectum, and no lymphatic anomaly has been reported in more than two parenchymal organs.

Cystic structures are observed in various orgnas on US and CT in our case. In Considering the patient's age, the possibility of neoplastic cyst was considered low. Since the lumen was intact on CT, the possibility of intraluminal lesions was considered low. Considering that the wall of the uterus is irregularly observed in connection with the septa of the cystic lesions, it was considered that the cyst originated from the wall of the organ rather than from the mesentery or peritoneal lesion. Therefore, GLA, adenomyosis and paramesonephric cyst were considered as differential diagnoses, which can't be ditinguished by imaging alone, and immunostaining tests are required. Becase GLA has been reported not only in young human patients, but also in older human patients, care should be taken not to misdiagnose GLA as metastatic cancer when cystic lesions occur in multiple organs (20, 21).

In human, the presence of chylous fluid in cysts with high triglycerides is pathognomonic for lymphangioma, and according to what has been reported so far, it has a common feature that small lymphocytes are mainly seen (22, 23). Therefore, fluid analysis can provide good information for diagnosing GLA. But depending on the location of the lymphatic anomaly, the fluid within the cyst may be clear transudate or chylous (24). Similarly, as a result of fluid analysis in a dog with lymphangioma in a previous report, it was considered as a transudate, and small lymphocytes were mainly observed (7). Fluid collections, whether chylorous or not, are mostly anechoic on ultrasound, and it is difficult to distinguish between simple fluid and chylous even on CT.

Lymphangiography can reveal leakage or obstruction of the lymphatic pathway. If a patient presents with pleural effusion or ascites, lymphangiography may be helpful to identify the lymphatic pathway for diagnosis and treatment planning (25). However, this is a major feature seen in CCLA, and in GLA, lymphangiography may not show any special findings.

Histopathology and immunostaining of LYVE-1, a marker associated with lymphatic vessels, must be performed to distinguish whether it is derived from lymphatic vessels vs. vascular endothelium (18). Caution is warranted while interpreting LYVE-1 and other Immunohistochemical findings. The H&E findings with the immunohistochemistry must be weighed together. LYVE-1, a CD44 homolog was thought to be restricted to lymphatics and did not stain endothelial cells, but subsequent studies showed non-lymphatic endothelial cells within hepatic sinusoids. Embryonic lymphangiogensis is under debate, but both vasculature and lymphatics are lined by endothelium. Other markers previously thought to be specific to lymphatics have been shown to lack specificity: VEGFR-3 has been seen in a subset of angiogenic vessels associated with pathologic conditions (26). Podoplanin is seen in smaller lymphatics lacking smooth muscle and not in high endothelial venules, and is also expressed in osteoblasts, renal podocytes and type I pulmonary alveolar cells. LYVE-1 has also been identified in hepatic sinusoids in normal livers and down regulated in sinusoids in some pathologic hepatic conditions (27). In our case, LYVE-1 staining was seen in blood filled vessels with intermediate staining (Figure 3C) and in empty endothelial lined vessels with intermediate to strong staining. These findings in conjunction with multiple empty cystic spaces seen in Figure 2 support a lymphatic origin.

In humans, the PIK3CA gene and NRAS gene are known to be associated with generalized lymphatic anomaly, but there is no study on the related genes in animals (15, 16). Therefore, in the present case, DNA was extracted and PCR was performed using FFPE tissue for NRAS gene sequencing, but the peak size was observed to be 44bp instead of 150, which was the base pairs of the target gene. This is considered as an amplification occurring by attaching primer dimers to each other, given that the same result was obtained both times and target gene was not detected. This may be because of deterioration due to oxidation of nucleic acid as the surface of the paraffin sections was exposed to the air, since PCR was performed more than 6 months after the paraffin-embedded sample was made. In addition, factors like formalin, tissue processing, sectioning and staining procedures, incubation at high temperature during the paraffin embedding process are also thought to have influenced DNA damage (28).

In humans, five out of six patients with lymphatic anomaly of the UB showed clinical signs of hematuria (29). To reduce the possibility of recurrence in most cases, partial cystectomy with complete resection rather than transurethral resection or laser ablation was performed as a treatment method. A patient with lymphatic anomaly in the uterine corpus showed clinical symptoms of back pain, leg edema, and abdominal distension, and was treated surgically through hysterectomy (30). In our case, there were no specific clinical symptoms, other than vomiting and lethargy. Since that there was no vomiting after debulking, it was considered to be due to an increase in abdominal pressure caused by cysts occupying most of the abdominal cavity.

The best treatment for lymphatic anomalies is surgical removal. However, since lymphangioma often includes deep tissue, complete resection may not be possible, and if only the superficial part is removed, the recurrence rate is approximately 20% in humans (29). If complete removal is not possible, medical treatment is available. In humans, sclerotherapy with OK-432, doxycycline, and bleomycin is the most commonly used alternative treatment for lymphangioma, because sclerotherapy is safe and has no serious side effects (10). Sclerotherapy destroys the vessel' endothelium, causing obstruction and fibrosis. Radiation therapy and sirolimus, which suppress the activity of the mTOR and promote lymphangiogenesis, have also been used as treatments in humans. However, there are few cases in which these treatments have been applied to dogs. Recently, there has been a case of canine lymphatic anomaly treated with sclerotherapy, electrochemotherapy, and radiation therapy (6) and a case of lymphangiosarcoma treated with toceranib, a tyrosine kinase inhibitor (15, 31).

Our study has several limitations. First, biopsy of the rectum was not performed. However, the US and CT showed the same pattern as the cysts in other organs, and since the same immunostaining results were obtained in all other five sites, the cyst observed in the rectum was also considered to be caused by GLA. Second, complete resection was not achieved in our case. Only debulking was performed because the cysts were so extensive that complete resection was impossible. In addition, sclerotherapy was not performed, because the cyst was multilobulated. Radiation therapy can be applied, but radiation hazards can occur in the heart and lungs, and since there is a case that showed a good prognosis without treatment other than debulking in humans, only debulking was performed in our case (14, 32). At 6 months follow-up, the size of the remaining cysts was maintained, and no clinical symptoms or specific findings were observed. Third, genetic testing didn't work properly. Further studies on GLA-related genes through fresh frozen tissue or blood are considered necessary. Fourth, fluid analysis of the cyst was not performed. However, It is considered that there was no significant effect on the diagnosis because the higher-level examination, such as biopsy and immunostaining, was performed. Finally, follow-up period was short. Thus, it is difficult to judge the effectiveness of the treatment and the prognosis of the GLA.

To the best of our knowledge, in veterinary medicine, this is the first case of lymphatic anomaly of the ovary, uterus, bladder, and rectum, and the first case of GLA involving more than two parenchymal organs. In the case of multiple cysts in multiple organs of the abdominal cavity in young dogs, GLA should be included in the differential diagnosis.

## Ethics statement

Ethical approval was not required for the study because the case report is a description of a clinical case. Written informed consent was obtained from the owners for the participation of their animals in this study.

## Author contributions

S-HP and J-HL drafted the manuscript. S-HP, J-HL, and HY contributed to case management. HY confirmed the cases and revised the final submission. ES performed the pathological studies. MK and J-iH performed the gene analysis. All authors contributed to the article and approved the submitted version.
